# Evaluation of three rapid diagnostic tests for the detection of human infections with *Plasmodium knowlesi*

**DOI:** 10.1186/1475-2875-13-60

**Published:** 2014-02-18

**Authors:** Deshka Foster, Janet Cox-Singh, Dayang SA Mohamad, Sanjeev Krishna, Pek P Chin, Balbir Singh

**Affiliations:** 1 Malaria Research Centre, Universiti Malaysia Sarawak, Kuching, Sarawak, Malaysia; 2 Stanford University, Stanford, USA; 3 University of St. Andrews, Scotland, UK; 4 Institute for Infection and Immunity, St. George’s, University of London, Cranmer Terrace, London SW17 0RE, UK; 5 Sarikei Hospital, Sarikei, Sarawak, Malaysia

**Keywords:** *Plasmodium knowlesi*, Malaria diagnostics, Rapid diagnostic tests

## Abstract

**Background:**

*Plasmodium knowlesi*, a malaria parasite of Southeast Asian macaques, infects humans and can cause fatal malaria. It is difficult to diagnose by microscopy because of morphological similarity to *Plasmodium malariae*. Nested PCR assay is the most accurate method to distinguish *P. knowlesi* from other *Plasmodium* species but is not cost effective in resource-poor settings. Rapid diagnostic tests (RDTs) are recommended for settings where malaria is prevalent. In this study, the effectiveness of three RDTs in detecting *P. knowlesi* from fresh and frozen patient blood samples was evaluated.

**Methods:**

Forty malaria patients (28 *P. knowlesi*, ten *P. vivax* and two *P. falciparum*) diagnosed by microscopy were recruited in Sarawak, Malaysian Borneo during a 16-month period. Patient blood samples were used to determine parasitaemia by microscopy, confirm the *Plasmodium* species present by PCR and evaluate three RDTs: OptiMAL-IT, BinaxNOW® Malaria and Paramax-3. The RDTs were also evaluated using frozen blood samples from 41 knowlesi malaria patients.

**Results:**

OptiMAL-IT was the most sensitive RDT, with a sensitivity of 71% (20/28; 95% CI = 54-88%) for fresh and 73% (30/41; 95% CI = 59-87%) for frozen knowlesi samples. However, it yielded predominantly falciparum-positive results due to cross-reactivity of the *P. falciparum* test reagent with *P. knowlesi*. BinaxNOW® Malaria correctly detected non-*P. falciparum* malaria in *P. knowlesi* samples but was the least sensitive, detecting only 29% (8/28; 95% CI = 12-46%) of fresh and 24% (10/41; 95% CI = 11-37%) of frozen samples. The Paramax-3 RDT tested positive for *P. vivax* with PCR-confirmed *P. knowlesi* samples with sensitivities of 40% (10/25; 95% CI = 21-59%) with fresh and 32% (13/41; 95% CI = 17-46%) with frozen samples. All RDTs correctly identified *P. falciparum-* and *P. vivax*-positive controls with parasitaemias above 2,000 parasites/μl blood.

**Conclusions:**

The RDTs detected *Plasmodium* in *P. knowlesi*-infected blood samples with poor sensitivity and specificity. Patients with *P. knowlesi* could be misdiagnosed as *P. falciparum* with OptiMAL-IT, *P. vivax* with Paramax-3 and more correctly as non-*P. vivax*/non-*P. falciparum* with BinaxNOW® Malaria. There is a need for a sensitive and specific RDT for malaria diagnosis in settings where *P. knowlesi* infections predominate.

## Background

Until recently only four types of *Plasmodium* (*Plasmodium falciparum, Plasmodium vivax, Plasmodium malariae* and *Plasmodium ovale*) were known to cause malaria in humans*.* However, a fifth species, *Plasmodium knowlesi,* has been identified as a cause of human malaria in almost all countries in Southeast Asia (recently reviewed [1]) and extending to the Nicobar and Andaman Islands in India [2]. In Malaysian Borneo, *P. knowlesi* is the main cause of admissions for malaria in certain hospitals, including Sarikei Hospital, and can lead to fatal infections [3-9].

*Plasmodium* species infections are typically diagnosed by microscopic examination of stained blood films, but there are limitations in sensitivity and specificity [10]. Nested PCR assays were developed to accurately distinguish between *Plasmodium* species. Molecular methods are the most sensitive detection method for malaria and can distinguish *P. knowlesi* from the morphologically similar *P. malariae*[11,12]. However, both microscopy and especially nested PCR assays require significant resources, such as specialized equipment, electricity and skilled technicians. PCR methods are not recommended for malaria diagnosis in resource-poor settings.

Malaria antigen-based rapid diagnostic tests (RDTs), mainly for the detection of *P. falciparum*, were developed for use in remote areas with limited facilities. A large number of RDTs are available for malaria diagnosis, including OptiMAL-IT produced by DiaMed, CA, USA [13,14], BinaxNOW® Malaria produced by Inverness Medical, FL, USA [15,16], and Paramax-3 malaria Pf/Pv/Pan from Zephyr Biomedical Systems, India [17,18]. The OptiMAL-IT test strip contains the antibody 17E4, which specifically detects *P. falciparum* lactate dehydrogenase (pLDH) and a pan-malaria antibody (19G4) that identifies pLDH from *P. vivax, P. malariae* and *P. ovale* as well as *P. falciparum.* BinaxNOW® Malaria is the only test approved by the US FDA and contains an antibody that detects *P. falciparum*-specific histidine rich protein 2 (HRP-2) and a pan-malaria antibody that detects *Plasmodium* aldolase, a glycolytic enzyme produced by all species of the parasite. The Paramax-3 test strip contains three antibody test lines as well as a control line: an antibody detecting *P. falciparum* HRP-2, similar to the BinaxNOW® Malaria test, a pan-malaria pLDH antibody, and an antibody specific for *P. vivax* LDH. None of the tests include *P. knowlesi-*specific reagents.

OptiMAL-IT, and another RDT under development [19], have been shown to detect *P. knowlesi* under experimental conditions*.* An imported case of human *P. knowlesi* from Sarawak to the Netherlands with 2% parasitaemia (84,000 trophozoites/μl) gave positive results for both the *P. falciparum*-specific LDH and pan-malarial LDH test lines, suggesting cross-reactivity between *P. knowlesi* LDH and the monoclonal antibody used in the test to detect *P. falciparum* LDH [20]. This cross-reactivity was first reported by McCutchan *et al.*[19], was also noted by Kawai *et al*. using *P. knowlesi*-infected Japanese macaque blood samples with OptiMAL-IT [21] and by Ong *et al.* using a *P. knowlesi*-positive blood sample from a patient with 0.2% parasitaemia [22].

BinaxNOW® Malaria has been used to detect *P. knowlesi* in several case reports with varying results [20,22-25]. In a case of *P. knowlesi* malaria imported into the Netherlands, BinaxNOW® Malaria but not OptiMAL-IT gave a positive result for non-*P. falciparum* malaria [20]. Similar results were reported in another case [23]. However, BinaxNOW® can also cross react and show falciparum-positive results, as observed with a *P. knowlesi*-infected patient with 0.2% parasitaemia [22]. False-negative results were also obtained using this test with two *P. knowlesi* malaria patients with parasitaemia of 0.0005 and 0.1% [24,25], suggesting that BinaxNOW® Malaria may be less sensitive than OptiMAL-IT.

None of the aformentioned studies has evaluated the Paramax-3 test with *P. knowlesi* samples. The objective of this study was to evaluate three RDTs, OptiMAL-IT, BinaxNOW® Malaria, and Paramax-3, in the detection of *P. knowlesi* infection from both fresh and frozen blood samples from knowlesi malaria patients.

## Methods

### Study sites

All consecutive malaria-positive patients, diagnosed by microscopy, were recruited following acquisition of informed consent at Sarikei Polyclinic and Sarikei Hospital in the town of Sarikei, Sarawak, Malaysian Borneo. The study was approved by the Malaysian Ministry of Health’s Medical Research and Ethics Committee.

### Collection and processing of fresh and frozen blood samples

A total of 40 malaria-positive blood samples were collected between March 2010 and July 2011: ten (seven *P. knowlesi* and three *P. vivax*) from Sarikei Polyclinic and 30 (21 *P. knowlesi*, seven *P. vivax* and two *P. falciparum*) from Sarikei Hospital. No mixed species infections were identified. Venous blood samples collected from these patients were used to make thick and thin blood films for verification of parasitaemia by microscopy, blood spots on filter paper for malaria species identification by nested PCR assays, and to evaluate RDTs.

The three RDTs were also evaluated using 44 frozen whole-blood samples collected from malaria patients with PCR-confirmed *P. knowlesi* (N = 41), *P. falciparum* (N = 3) and *P. vivax* (N = 1), admitted to Sarikei and Sibu Hospitals in the two years prior to the start of this study and from those recruited during a previous study at Kapit Hospital [26]. Whole-blood samples, stored at -80°C, were thawed and used to evaluate the RDTs as recommended.

### Analysis of samples by microscopy

Thick blood films from samples acquired at Sarikei Polyclinic and Hospital were allowed to dry overnight, then stained with 3% Giemsa for 45 minutes. Thin blood films were fixed with methanol and stained with 10% Giemsa for 30 minutes. Parasitaemia was later determined by an experienced microscopist. Parasitaemia was calculated as the number of parasites per μl of blood for each sample by using each patient’s actual white blood cell (WBC) count, and by counting up to 500 WBCs in thick blood films and the corresponding number of malaria parasites.

### Analysis of samples by nested PCR assay

DNA was extracted from the blood spots on filter paper using the Instagene method [26]. All samples were initially examined by PCR assay using the *Plasmodium* genus-specific primers rPLU1 and rPLU5 for the Nest 1 amplification, which targets the small sub-unit ribosomal RNA gene of the *Plasmodium* genus [21]. Reaction mixtures for no more than ten samples were prepared and processed at one time and a positive and negative control were processed with each batch of samples to maintain high-quality control.

In order to identify the malaria species present in each sample, Nest 1 amplification products were screened using Nest 2 species-specific primers, as described previously [12,26,27] for each of the five species of malaria known to infect humans: *P. falciparum, P. vivax, P. malariae, P. ovale* and *P. knowlesi*. For the species-specific primer pairs for *P. falciparum, P. vivax, P. malariae* and *P. ovale* (rFAL1/rFAL2, rVIV1/rVIV2, rMAL1/rMAL2, and rOVA1/rOVA4) the annealing temperature was 58°C, and for the *P. knowlesi*-specific primer pair (kn1f and kn3r) the annealing temperature was 62°C. The Nest 2 products were analysed using agarose gel (2.5%) electrophoresis and Sybr green® staining (1x concentration for 30 minutes), viewed via UV transilluminator, photographed for documentation and recorded.

### Analysis of samples by RDTs

The three antigen-based RDTs, OptiMAL-IT, BinaxNOW® Malaria, and Paramax-3, were used according to the manufacturers’ instructions. Fresh blood samples from malaria patients were processed within three hours of collection, and immediately when using thawed frozen blood samples. Thawed samples were processed as for fresh blood. OptiMAL-IT requires 10 μl of blood and takes 20 minutes to complete, BinaxNOW® Malaria requires 15 μl of blood and takes 10–15 minutes to complete, and Paramax-3 requires 5 μl of blood and takes 15 minutes to complete. Tests were interpreted as successful when the control band was positive.

### Analysis of results of RDTs

The sensitivity of each RDT was calculated with PCR results as the reference standard. The 95% confidence interval (CI) for each test was calculated based on the test sensitivity and number of tests performed using 1.96 as the standard normal deviate (using the formula: 95% CI = Sensitivity ± 1.96 × standard error of the test) [28].

## Results

RDTs were evaluated against 40 microscopy-positive fresh blood samples (identified by nested PCR assay as 28 *P. knowlesi*, two *P. falciparum* and ten *P. vivax*), along with 44 frozen whole-blood malaria samples (41 *P. knowlesi*, two *P. falciparum* and one *P. vivax* by nested PCR assay). All RDTs were successful in that the control line was positive on all tests performed.

In total, 28 OptiMAL-IT and BinaxNOW® Malaria and 25 Paramax-3 tests were performed on *P. knowlesi* field isolates (median parasitaemia = 9,131 parasites/μl; range = 159–911,616 parasites/μl blood) and 41 of each RDT were performed using frozen *P. knowlesi* samples (median parasitaemia = 1,297 parasites/μl; range = 10–188,384 parasites/μl blood).

OptiMAL-IT was the most sensitive of the three RDTs evaluated, with a sensitivity of 71% (20/28; 95% CI = 54-88%) and 73% (30/41; 95% CI = 59-87%) for fresh and frozen knowlesi malaria samples, respectively (Tables 1 and 2). However, the test was not specific and *P. knowlesi* samples cross-reacted with the *P. falciparum* LDH test reagent in 18 of the 20 fresh samples identified. Only two of the fresh *P. knowlesi* samples were identified as non-*P. falciparum* malaria using OptiMAL-IT.

**Table 1:** **Rapid diagnostic test results for *Plasmodium knowlesi *** **fresh blood from field isolates**

***P. knowlesi*** **parasitaemia (parasites/μl)**	**OptiMAL-IT**		**BinaxNOW® Malaria**		**Paramax-3**	
		**Number of samples tested**		**Number of samples tested**		**Number of samples tested**
**>5,000**	Pf-positive (Pf and Pan-positive)	13	Pf-positive	0	Pf-positive (Pf and Pan-positive)	1
	Pan-positive (Pv/Pm/Po) only	1	Pan-positive (Pv/Pm/Po) only	8	Pv-positive (Pv and Pan-positive)	7
					Pan-positive (Pm/Po) only	0
	Negative	4	Negative	10	Negative	9
**1,001-5,000**	Pf-positive (Pf and Pan-positive)	4	Pf-positive	0	Pf-positive (Pf and Pan-positive)	0
	Pan-positive (Pv/Pm/Po) only	1	Pan-positive (Pv/Pm/Po) only	0	Pv-positive (Pv and Pan-positive)	1
					Pan-positive (Pm/Po) only	1
	Negative	2	Negative	7	Negative	3
**501-1,000**	Pf-positive (Pf and Pan-positive)	0	Pf-positive	0	Pf-positive (Pf and Pan-positive)	0
	Pan-positive (Pv/Pm/Po) only	0	Pan-positive (Pv/Pm/Po) only	0	Pv-positive (Pv and Pan-positive)	0
					Pan-positive (Pm/Po) only	0
	Negative	2	Negative	2	Negative	2
**1-500**	Pf-positive (Pf and Pan-positive)	1	Pf-positive	0	Pf-positive (Pf and Pan-positive)	0
	Pan-positive (Pv/Pm/Po) only	0	Pan-positive (Pv/Pm/Po) only	0	Pv-positive (Pv and Pan-positive)	0
					Pan-positive (Pm/Po) only	0
	Negative	0	Negative	1	Negative	1
**Total**	Pf-positive (Pf and Pan-positive)	**18**	Pf-positive	**0**	Pf-positive (Pf and Pan-positive)	**1**
	Pan-positive (Pv/Pm/Po) only	**2**	Pan-positive (Pv/Pm/Po) only	**8**	Pv-positive (Pv and Pan-positive)	**8**
					Pan-positive (Pm/Po) only	**1**
	Negative	**8**	Negative	**20**	Negative	**15**
**Total tested**		**28**		**28**		**25**
**Total positive**		**20**		**8**		**10**
**Sensitivity**		**71%**		**29%**		**40%**
(95% CI)		(54 – 88%)		(12 – 46%)		(21 – 59%)

**Table 2:** **Rapid diagnostic test results from frozen *Plasmodium knowlesi *** **blood samples**

***P. knowlesi *** **parasitaemia (parasites/μl)**	**OptiMAL-IT**		**BinaxNOW® Malaria**		**Paramax-3**	
		**Number of samples tested**		**Number of samples tested**		**Number of samples tested**
**>5,000**	Pf-positive (Pf and Pan-positive)	8	Pf-positive	0	Pf-positive (Pf and Pan-positive)	0
	Pan-positive (Pv/Pm/Po) only	0	Pan-positive (Pv/Pm/Po) only	5	Pv-positive (Pv and Pan-positive)	7
					Pan-positive (Pm/Po) only	0
	Negative	0	Negative	3	Negative	1
**1,001-5,000**	Pf-positive (Pf and Pan-positive)	12	Pf-positive	0	Pf-positive (Pf and Pan-positive)	0
	Pan-positive (Pv/Pm/Po) only	1	Pan-positive (Pv/Pm/Po) only	2	Pv-positive (Pv and Pan-positive)	4
					Pan-positive (Pm/Po) only	0
	Negative	0	Negative	11	Negative	9
**501-1,000**	Pf-positive (Pf and Pan-positive)	4	Pf-positive	0	Pf-positive (Pf and Pan-positive)	0
	Pan-positive (Pv/Pm/Po) only	0	Pan-positive (Pv/Pm/Po) only	2	Pv-positive (Pv and Pan-positive)	1
					Pan-positive (Pm/Po) only	0
	Negative	4	Negative	6	Negative	7
**1-500**	Pf-positive (Pf and Pan-positive)	4	Pf-positive	0	Pf-positive (Pf and Pan-positive)	0
	Pan-positive (Pv/Pm/Po) only	1	Pan-positive (Pv/Pm/Po) only	1	Pv-positive (Pv and Pan-positive)	1
					Pan-positive (Pm/Po) only	0
	Negative	7	Negative	11	Negative	11
**TOTAL**	Pf-positive (Pf and Pan-positive)	**28**	Pf-positive	**0**	Pf-positive (Pf and Pan-positive)	**0**
	Pan-positive (Pv/Pm/Po) only	**2**	Pan-positive (Pv/Pm/Po) only	**10**	Pv-positive (Pv and Pan-positive)	**13**
					Pan-positive (Pm/Po) only	**0**
	Negative	**11**	Negative	**31**	Negative	**28**
**Total tested**		**41**		**41**		**41**
**Total positive**		**30**		**10**		**13**
**Sensitivity**		**73%**		**24%**		**32%**
(95% CI)		(59 – 87%)		(11 – 37%)		(17 – 46%)

BinaxNOW® Malaria showed the lowest sensitivity, detecting only 29% (8/28; 95% CI = 12-45%) of fresh and 24% (10/41; 95% CI = 11-37%) of frozen samples. This test was negative for all ten *P. knowlesi* fresh blood samples with parasitaemia <5,000 parasites/μl and also tested negative for 56% (10/18) of fresh samples with parasitaemia >5,000 parasites/μl (Table 1). However, with the BinaxNOW® Malaria test, which detects *P. falciparum*-specific histidine rich protein 2 (HRP-2), all positive results for *P. knowlesi* cases were correctly identified as non-*P. falciparum* malaria infections.

The Paramax-3 test also had low sensitivities of 40% (10/25; 95% CI = 21-59% ) and 32% (13/41; 95% CI = 18-46%) for fresh and frozen *P. knowlesi* samples, respectively. Of the ten Paramax-3 tests that yielded positive results with fresh blood samples, one indicated a *P. falciparum* infection, one indicated a non-*P. falciparum*, non-*P. vivax* result and the remaining eight gave results indicating *P. vivax* infections (Table 2).

*Plasmodium vivax* (N = 10) and *P. falciparum* (N = 2) samples from Sarikei Hospital and Polyclinic were collected for testing with RDTs as positive controls. All three RDTs gave positive results accurate for the species present when the parasitaemias were above 2,000 parasites/μl blood (range: 240–23,000 parasites/μl). The RDTs did not detect samples with *P. vivax* (N = 2) or *P. falciparum* (N = 1) parasitaemia less than 2,000 parasites/μl. All frozen *P. vivax* (N = 1) and *P. falciparum* (N = 2) positive controls yielded positive RDT results appropriate for the species. The parasitaemia for these samples was relatively high (19,000-30,000 parasites/μl).

## Discussion

Among the RDTs tested in this study, the OptiMAL-IT test was the most sensitive for *P. knowlesi*-infected blood samples. However, the test was not specific and the majority of *P. knowlesi* samples were identified as *P. falciparum* by this test due to antibody cross-reactivity, as noted in previous studies [19-22]. BinaxNOW® Malaria was found to be the least sensitive of the three RDTs assessed, but there was no cross-reactivity observed between the *P. falciparum* antibody for *P. falciparum*-HRP-2 and *P. knowlesi* samples. In this study, all positive results attained using this test with *P. knowlesi*-confirmed samples correctly indicated a non-*P. falciparum* malaria infection. The Paramax-3 test showed low sensitivity and cross-reactivity between the *P. vivax* LDH-detecting antibody and *P. knowlesi*. This observation has been noted in several other single case reports using different tests which also contain a *P. vivax* LDH-detecting antibody [19,23,24].

The sensitivity of detecting *P. knowlesi* in blood samples with all three RDTs assessed in this study was significantly lower than that reported for other *Plasmodium* species. For example, the sensitivity of detection of *P. falciparum* using OptiMAL-IT has been reported as 95.3% (100% for >500 parasites/μl and 72% for 50 parasites/μl) and 96% for *P. vivax* malaria infections [14]. For BinaxNOW® Malaria, the sensitivity of detection for *P. falciparum* has been reported as 95.3% (99.7% for >5,000 parasites/μl and 53.9% for 100 parasites/μl or fewer) and 68.9% for *P. vivax* malaria infections [16]. For Paramax-3, the sensitivity and specificity of detection for both *P. falciparum* and *P. vivax* malaria infections is reported as 100% in an in-house study of 251 samples [29]. Although the number of positive controls conducted in this study was relatively low, none of the RDTs used in this study detected *P. vivax* or *P. falciparum* infections in fresh blood samples with parasitaemias less than 2,000 parasites/μl.

One limitation of this study is the relatively low numbers of fresh samples tested. To strengthen the results from fresh samples, frozen blood samples were also included. The sensitivity of detection of knowlesi malaria infections with the RDTs tested were fairly similar using fresh *versus* frozen samples. Freeze-thawing and the storage of blood at low temperatures can accelerate deterioration of antigen activity, although it is also possible that target antigens are more accessible in freeze-thawed samples and may actually improve the sensitivity of RDTs [30].

A recently published paper from Sabah, Malaysian Borneo presents the use of two different RDTs, First Response™, which detects pan-*Plasmodium* LDH and Pf-specific HRP-2, and ParaHIT™, which detects pan-*Plasmodium* aldolase and Pf-specific HRP-2 [7]. A total of 129 *P. knowlesi* patient samples were studied, only 34 of whom were enrolled in the study prior to treatment, while the remainder were referred from district hospitals where they had already received anti-malarial treatment [7]. The findings of this study indicated a sensitivity of 74% for the pLDH component of the First Response™ RDT, which is similar to that observed in the current study with OptiMAL-IT (71 and 73% sensitivity with fresh and frozen samples, respectively), and higher than that observed with the Paramax-3 test (40 and 32% sensitivity), both of which also detect pLDH. In the current study as well as the one in Sabah, the RDTs with the pan-aldolase component had the lowest sensitivity of detection of *P. knowlesi* samples; 29% with the ParaHIT™ test [7], and 29% with fresh and 24% with frozen samples using the BinaxNOW® Malaria RDT.

RDTs cost between 10 and 15 Malaysian Ringgit (US$3.17-4.80) per test when purchased at a dispensary in Malaysia. Although when purchased in bulk for malaria control programs this cost tends to be significantly reduced, microscopy is still the most affordable diagnostic tool and costs just one Ringgit (US$0.30) per patient to screen for malaria. The cost of nested PCR assay is comparable to RDTs per patient sample, and although PCR assay is significantly more sensitive and specific than microscopy, this technique requires specialized equipment, electricity supply and training, and is not suitable for resource-poor settings. RDTs confer the advantages of speed (all types used in this study took 20 minutes or less to conduct), minimal training and ease of use, and do not require electricity or any specific hardware. However, currently available RDTs lack sensitivity and specificity compared to microscopy and PCR-based methods for all *Plasmodium* species infections, especially *P. knowlesi*. The development of loop-mediated isothermal amplification (LAMP) assays combine the sensitivity and specificity of PCR with low cost, low technology and rapid results. LAMP-based tests for malaria diagnosis that include reagents specific for *P. knowlesi* are under development and may be useful for resorce-poor settings [31,32].

In areas with relatively low malaria prevalence such as Sarawak, the cost of RDTs, even if sensitive and specific, would likely outweigh the benefit. To understand this in practical terms, consider, for example, the case of Julau Health Clinic, which is a small, rural health clinic in Sarawak surveyed as part of the current study. The prevalence of malaria at this clinic during a five-month study period in which 108 febrile patients whose clinical presentations were suggestive of malaria were screened using nested PCR assay was 0.2% (Foster *et al.*, unpublished data). As such, it would have cost between MYR 1,080 and 1,620 (US$342-832) to perform RDTs for these 108 query malaria patients and only two were positive.

This study confirms that the RDTs evaluated are not adequately sensitive for use in the diagnosis of *P. knowlesi*. Also, *P. knowlesi* cross-reacted with *P. falciparum* and *P. vivax* LDH antibodies used in two of the three commercially available RDTs tested, resulting in misdiagnosis of malaria species in an area where human *P. knowlesi* infections are prevalent. Since not all species of malaria warrant the same level of medical care, misidentification can result in mismanagement, especially when the potentially severe knowlesi malaria is misdiagnosed as vivax malaria. Because *P. knowlesi* is morphologically similar to *P. malariae* and *P. falciparum,* it is also misdiagnosed by microscopy [10,23,24]. However microscopy should not be replaced by RDTs in areas where *P. knowlesi* occurs until the sensitivity, specificity and costs are comparable.

## Conclusions

The sensitivity of detection of *P. knowlesi* by the three RDTs evaluated is low compared with microscopy. Cross-reactivity is common between *P. knowlesi-*infected blood and both the *P. falciparum*-detecting antibody used in the OptiMAL-IT test and the *P. vivax*-detecting antibody used in the Paramax-3 test. As such, a patient with knowlesi malaria may be diagnosed as *P. falciparum* by OptiMAL-IT, as *P. vivax* by Paramax-3 and as non-*P.vivax* and non-*P. falciparum* by BinaxNOW® Malaria. Until more sensitive RDTs are developed that can distinguish *P. knowlesi* from *P. falciparum* and *P. vivax*, serious consideration should be taken before using RDTs for the diagnosis of malaria in settings where *P. knowlesi* is the predominant species.

## Competing interests

The authors declare that they have no competing interests.

## Authors’ contributions

BS, JCS, SK and DF were involved in study conception and design, procurement of RDTs and writing the manuscript. DF was involved in data collection and analysis, and drafting the manuscript. PPC assisted with data collection and analysis and DSAM assisted with laboratory data analysis. BS was involved in training of personnel and oversaw the project throughout. All the authors have read and approved the manuscript.
